# Tracing Mobile DNAs: From Molecular to Population Scales

**DOI:** 10.3389/fpls.2022.837378

**Published:** 2022-02-01

**Authors:** Wenwen Fan, Ling Wang, Jie Chu, Hui Li, Eun Yu Kim, Jungnam Cho

**Affiliations:** ^1^National Key Laboratory of Plant Molecular Genetics, CAS Center for Excellence in Molecular Plant Sciences, Shanghai Institute of Plant Physiology and Ecology, Chinese Academy of Sciences, Shanghai, China; ^2^University of Chinese Academy of Sciences, Beijing, China; ^3^CAS-JIC Centre of Excellence for Plant and Microbial Science, Chinese Academy of Sciences, Shanghai, China

**Keywords:** transposon, long terminal repeat (LTR) retrotransposon, retrotransposition, ALE-seq, mobilome-seq, long-read sequencing, droplet digital PCR (ddPCR)

## Abstract

Transposable elements (TEs, transposons) are mobile DNAs that are prevalent in most eukaryotic genomes. In plants, their mobility has vastly contributed to genetic diversity which is essential for adaptive changes and evolution of a species. Such mobile nature of transposon has been also actively exploited in plant science research by generating genetic mutants in non-model plant systems. On the other hand, transposon mobilization can bring about detrimental effects to host genomes and they are therefore mostly silenced by the epigenetic mechanisms. TEs have been studied as major silencing targets and acted a main feature in the remarkable growth of the plant epigenetics field. Despite the importance of transposon in plant biology and biotechnology, their mobilization and the underlying mechanisms are largely left unanswered. This is mainly because of the sequence repetitiveness of transposons, which makes their detection and analyses difficult and complicated. Recently, some attempts have been made to develop new experimental methods detecting active transposons and their mobilization behavior. These techniques reveal TE mobility in various levels, including the molecular, cellular, organismal and population scales. In this review, we will highlight the novel technical approaches in the study of mobile genetic elements and discuss how these techniques impacted on the advancement of transposon research and broadened our understanding of plant genome plasticity.

## Introduction

Transposable elements (TEs or transposons) are stretches of DNA that move around the genomes and are ubiquitous in most eukaryotic genomes (Feschotte, 2008; Lisch, 2012; Chuong et al., 2017). Particularly, the genomes of major food crops such as barley, wheat and maize contain myriads of transposons making up more than 80% of their genomes (Tenaillon et al., 2010). Among the diverse types of transposons, the long terminal repeat (LTR) retrotransposon is the predominant type of TEs in most plant genomes (Casacuberta and Santiago, 2003; Grandbastien, 2015; Cho, 2018; Satheesh et al., 2021) and thus will be the main focus of this review. The mobilization of an LTR retrotransposon is mediated by the reverse transcription of TE mRNAs to cDNAs (also referred to as extrachromosomal DNA, ecDNA), which happens in virus-like particles (VLPs) and is followed by the insertion to new genomic positions by the integrase (Cho et al., 2019; Satheesh et al., 2021). Due to the mobile nature of transposons and thereby potential danger of genomic instability, they are subject to the host genomes’ epigenetic silencing pathways, including chromatin modification and DNA methylation (Slotkin and Martienssen, 2007; Matzke and Mosher, 2014). On the other hand, transposon is one of the major sources of genetic diversity, which is critical for evolution and adaptive changes of plants (Lisch, 2012; Dubin et al., 2018). Besides, TEs have been actively exploited in the plant science field as useful mutagenic reagents. For example, *Tos17* in rice is specifically activated by *in vitro* tissue culture and the resulting random insertional mutants tagged with *Tos17* are important genetic resources in the rice functional genomics (Hirochika et al., 1996; Hirochika, 2010). Similarly, *Tnt1* was used to generate genetic mutants in *Medicago truncatula*, *Brachypodium distachyon*, and *Glycine max* (D’Erfurth et al., 2003; Tadege et al., 2008; Revalska et al., 2011; Cui et al., 2012; Nandety et al., 2020), and the maize *Ac/Ds* DNA transposon system was used as a functional genomics tool in *Arabidopsis*, *Oryza sativa*, and *Glycine max* (Long et al., 1993; Mathieu et al., 2009; Wang et al., 2013). Despite the vast importance of transposons, little is known about the regulatory mechanisms of their mobilization, which is largely because of the lack of experimental methods that can detect the transposition events with sufficient sensitivity and precision.

**FIGURE 1:**
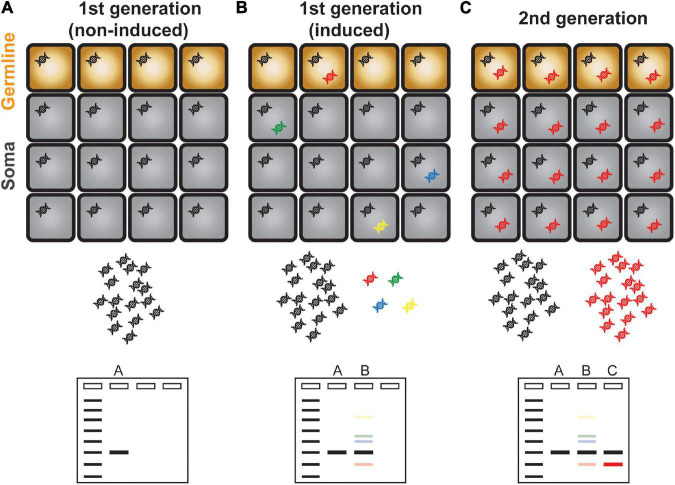
Schematic illustration of transposon display. **(A)** A single copy TE is present evenly in all cells and is represented as a single band in a transposon display experiment. **(B)** Activation and mobilization of a TE gave rise to new and additional copies inserted in different genomic positions as represented in different colors. Because of the scarcity of newly copied DNA, transposon display method is unable to amplify these DNAs which are illustrated as faint bands. **(C)** The new TE copy that mobilized in germline cells is inherited to the next generation. The transgenerationally maintained new TE DNA can be amplified efficiently and is visible as a discrete band in a gel electrophoresis.

It is well documented that transposons can be transcriptionally activated by the environmental challenges and at specific cell types and developmental stages (Martínez and Slotkin, 2012; Cho, 2018; Cho et al., 2019). However, the mobilization of activated transposons hardly happens likely because of complex regulation at the post-transcriptional steps (Hung and Slotkin, 2021; Kim et al., 2021b). Owing to the scarcity of transposition events and technical difficulty to detect it, it has been challenging to study transposon mobilization. In the past, transposon insertion was inferred by phenotypic abnormalities caused by deleterious mutations of a gene disrupted by TE integration. For example, some of the epigenetic recombinant inbred lines (epiRILs) generated from the *met1* mutant in *Arabidopsis* exhibited various abnormal phenotypes, which were associated with gene disruption caused by the transposition of *Evade* retroelement (Mirouze et al., 2009; Reinders et al., 2009; Reinders and Paszkowski, 2009). A PCR-based technique called transposon display (TD) and its derivative methods are usually the experimental approaches of choice to detect and locate new insertions of a transposon of interest (Kim et al., 2021a). Briefly, the adapter with known sequence is ligated to the restriction enzyme-digested DNA ends. PCR amplification by the specific sequences of the adapter and transposon ends yields amplicons containing the genomic regions flanking the transposon of interest. Although TD is an efficient and versatile method to study transposition events, it has certain fundamental limitations; for instance, transposon of high copy number is difficult to be amplified and hardly detected for new insertions. In addition, TD requires prior knowledge of TE sequences and thus relies on the quality of TE annotation. Most importantly, TD can only reveal the insertions that are meiotically inherited and fixed in the genomes, thus is not able to detect transpositions in real time and those happened in somatic cells (Figure 1). Over the last several years, there have been significant efforts to unveil the landscape of transpositions in the plant genomes by developing novel experimental methods. These innovative approaches reveal the mobilomes at varying scales from molecular to population levels. In this review, we will introduce and discuss the up-to-date experimental techniques tracing mobile DNAs in the plant genomes.

### Molecular Level

**FIGURE 2:**
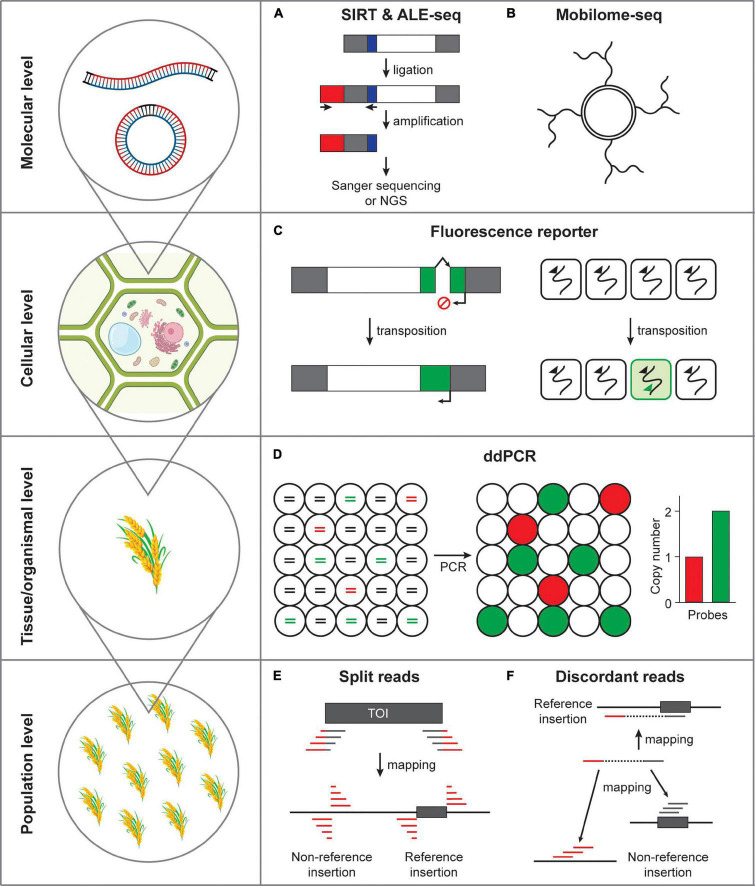
Methods detecting transposon mobility at varying scales. **(A)** Activated retrotransposon gives rise to extrachromosomal DNAs in both linear and circular forms. SIRT and ALE-seq are the methods that can detect linear DNA intermediates of retroelements. Gray box, long terminal repeats; white box, open reading frame; red box, adapter; blue box, PBS. **(B)** Extrachromosomal circular DNAs can be amplified by the isothermal strand displacement DNA polymerization. **(C)** Fluorescence reporter retrotransposition system can specifically label the cells in which transposition occurred. Green box, fluorescence reporter gene; arrows, transcriptional start sites; broken arrow, intron. **(D)** Transposon mobilization at tissue or organismal level can be assessed by ddPCR. Fragmented DNAs are randomly distributed in separate droplets and independently amplified. Lines in circles represent fragmented genomic DNA colored for different PCR templates. Red, a single-copy reference gene; green, transposon of interest; black, non-templated DNA. **(E)** The split reads method uses the reads tagged with transposon sequences that map to the insertion positions. TOI, transposon of interest; gray box, transposon; gray line, transposon sequence; red line, flanking sequence of transposon. **(F)** The discordant reads method uses the paired-end reads, which map to transposon from one side and to a distant genomic site from the other side of a read pair.

The mobilization cycle of an LTR retrotransposon consists of transcription, reverse transcription, and integration to new genomic positions. Since the direct detection of transposon integration is relatively more challenging, the DNA intermediate which is the final product of reverse transcription reaction and the direct target of integration has been studied to infer the transposon mobility. In this section, the cutting edge methods detecting the DNA intermediates of LTR retrotransposons will be highlighted (Figures 2A,B).

#### Detection of Linear ecDNA

The reverse transcription reaction of transposon gives rise to linear extrachromosomal DNAs (eclDNAs) and it is the linear form of ecDNAs that is capable of integrating to genomic DNA (Cho et al., 2019; Wang et al., 2021a,b). As an attempt to detect eclDNA, Griffiths et al. (2018) established a method named sequence-independent retrotransposon trapping (SIRT). SIRT employs the adapter ligation to the end of eclDNAs and specific amplification targeted to the conserved primer-binding site (PBS) sequence, which is located immediately after the upstream LTR. Using this method a novel family of LTR retrotransposon named *DODGER* was identified in the *Landsberg erecta* ecotype of *Arabidopsis* mutated with *MET1* (Griffiths et al., 2018). Unfortunately, SIRT exhibited limited robustness when tested in crop genomes, presumably because of the large size of the crop genomes and abundance of transposon-related sequences. An improved method was then developed named amplification of LTR extrachromosomal DNA followed by sequencing (ALE-seq), which is able to detect the LTRs of crop genomes with larger size (Cho et al., 2019). ALE-seq uses two primers specific to sequences of the adapter and PBS in two separate reactions: *in vitro* transcription and reverse transcription. Using this novel method, Cho et al. (2019) identified a new *Copia*-family LTR retrotransposon *Go-on* in the heat-stressed rice plants. Importantly, the ALE-seq method is particularly useful in non-reference crop species because the final amplicon product can reveal the full-length sequences of the LTR region. Such reference- and annotation-free approach was successfully tested in tomato pericarp samples and identified a novel *Gypsy*-family retroelement *Fruit-Induced RetroElement* (*FIRE*) (Cho et al., 2019). Although ALE-seq is sensitive enough to identify eclDNAs from crop genomes, it can only sequence the 5′ LTR regions and it is desired to further improve this method to cover full range of a TE. Altogether, ALE-seq is a versatile, efficient and high-throughput method identifying active LTR retroelements in crop genomes.

#### Detection of Circular ecDNA

Two LTRs of eclDNAs are bound by the integrases and homodimerization of integrases place two ends of an eclDNA close next to each other, which is then recognized as a DNA double-strand break by the cellular DNA damage response pathways (Møller et al., 2015, 2016; Lanciano et al., 2017). The homologous recombination and non-homologous end joining pathways repair the LTR-LTR gap, resulting in single-LTR and double-LTR extrachromosomal circular DNAs (eccDNAs), respectively (Lanciano et al., 2017). As a by-product of an activated LTR retrotransposon (albeit incapable of integration) eccDNA is considered to represent active TE mobility. Lanciano et al. (2017) established an experimental method called mobilome-seq that specifically sequences circular DNAs including retrotransposon-derived eccDNAs. The mobilome-seq procedure first initiates with digestion of linear DNA (mostly derived from genomic DNA) and randomly amplifying the remaining circular DNA by the isothermal stand displacement amplification (i.e., rolling circle amplification, Figure 2B). Unlike ALE-seq, mobilome-seq has additional advantage that can sequence full-length retroelement; however, it is also important to note that it reads sequences derived from organellar circular DNAs requiring additional filtering steps to remove them, which compromises the sequencing efficiency (Lanciano et al., 2017; Satheesh et al., 2021). Nonetheless, mobilome-seq can be a useful approach to investigate active retroelements because it requires relatively low sequence coverage, which can be particularly useful to studies using rare plant materials and samples with limited availability. For example, Lanciano et al. (2017) discovered a *PopRice* retrotransposon family that becomes active in the rice endosperm. In addition, Thieme et al. (2017) identified *Houba*, a *Copia*-like retrotransposon in rice, that was activated by the treatment of chemical inhibitors of RNA Polymerase II and DNA methylation. Moreover, Esposito et al. (2019) found that *nightshade*, a *Copia/Ale* retrotransposon in potatoes, produces large amount of eccDNAs in non-stressed plants, while under the cold stress condition is no longer active, presumably because of the hypermethylation induced by cold stress. More recently, mobilome-seq revealed that *Onsen*, a *Copia*-like retrotransposon specifically activated in the heat-stressed *Arabidopsis* plants, produces eccDNAs mostly from two copies, *AT1G11265* and *AT5G13205* (Roquis et al., 2021). In summary, mobilome-seq is a useful method detecting the retrotransposon mobility by sequencing eccDNAs.

#### Long-Read Sequencing

One of the challenges in the study of transposon is that TE sequences are repetitive in genomes and thus cause serious ambiguity in their analysis. This is particularly more troublesome when analyzing short-read sequencing data. Recently, the long-read sequencing technologies advanced remarkably and greatly improved the accuracy of transposon sequence analysis. For example, in the recent work of Panda and Slotkin, Oxford Nanopore Technology (ONT) sequencing was tested in the DNA methylation-deficient mutants of *Arabidopsis*, which significantly improved the quality of TE annotation (Panda and Slotkin, 2020). In an independent work by Lee et al. (2020) the ONT method was tested in the VLP fraction collected from the epigenetic mutants of *Arabidopsis*. This allowed direct identification of active transposable elements in their full lengths and also revealed diverse forms of DNA intermediates (Lee et al., 2020). Overall, the long-read sequencing technology is apparently a game-changer in the field of transposon research and highly expected to unveil the hidden aspects of transposon mobilization which was previously unable to be studied.

### Cellular Level

In the previous section, we focused on the methods detecting the DNA intermediates produced from active LTR retrotransposons which could be used as a proxy of TE mobility. It is important to note that the presence of DNA intermediates can be a good indication of TE activation; however, it does not necessarily represent transposition events directly. While in plants there has not been any robust methods detecting transposition events at the cellular level so far, the transposition reporter system used in humans and yeast has served as a standard method assessing transposon mobility. In this section, the transposition reporter assay systems revealing transposon insertion at the cellular level will be introduced (Figure 2C).

Retrotransposition reporter system was first suggested in yeast using the *Ty* retroelement *TyH3* that includes an intron fragment (Boeke et al., 1985). Heidmann et al. (1988) had later developed an improved version using the *neo* (neomycin phosphotransferase) gene cassette (neoRT). In this system, the neo gene is disrupted by an artificial intron containing polyadenylation signals, thereby the functional neo proteins can be produced only from the transposed intron-free DNA (Heidmann et al., 1988). This method allows for determination of transposition efficiency when cells are grown in the selective G418-containing media (Heidmann et al., 1988). Similar methods have been developed to study mobilization of other types of TEs including the intracisternal A-type particles (IAPs) in mice and the long interspersed elements (LINEs) in *Drosophila* and human cells (Heldmann and Heidmann, 1991; Jensen and Heidmann, 1991; Tchenio et al., 1993; Maestre et al., 1995; Esnault et al., 2000). Further improvement of retrotransposition assay system was attempted by Moran et al. (1996) by developing the reporter cassette consisting of an antisense copy of neo gene incorporated in two human L1 elements (L1.2 and LRE2) in a cultured human cell line (Rangwala and Kazazian, 2009). In addition, other alternative methods have also been developed by employing blasticidin S deaminase, his3 auxotrophic marker and a lacZ colorimetric indicator (Curcio and Garfinkel, 1991; Tchenio and Heidmann, 1992; Goodier et al., 2007). However, such intron-containing reporter systems had some drawbacks that retrotransposition assay is dependent on antibiotics resistance and assessed by counting colonies, which usually takes long time and has relatively low throughput. Recently, innovations to this classical method have been made by replacing the antibiotics resistance genes to visual fluorescence (Ostertag et al., 2000) and bioluminescence genes (Xie et al., 2011), which dramatically increases the sensitivity and throughput, enabling large-scale screening experiments. In summary, the retrotransposition assay systems have been widely used to determine the transposition rate of a retroelement mostly in non-plant systems. Introducing such system to the plant systems will enable single-cell detection of transposition and greatly improve our understanding of transposon mobilization.

### Tissue/Organism Level

#### Droplet Digital PCR

The retrotransposition reporter assay system described in the previous section can be potentially useful for cell- and tissue-level detection of transposition events. The synthetic artificial retrotransposon mobility assay is powerful because it enables direct visualization of transposition; however, such transgenic approach can be challenging in many non-reference plant species. Determination of copy number changes of an endogenous TE can be one of the easiest alternative methods to assess transpositional activity. It is worth noting, however, that the logarithmic quantitative real-time PCR analysis is difficult to measure subtle differences of copy number (Bubner and Baldwin, 2004; Bubner et al., 2004; Fan and Cho, 2021). Droplet digital PCR (ddPCR) is a far more accurate and sensitive technique that allows for digital measurement of DNA copy number (Hindson et al., 2013; Doi et al., 2015; Campomenosi et al., 2016; Głowacka et al., 2016; Fan and Cho, 2021). The ddPCR experiment performs DNA amplification in thousands of nanoliter-scale droplets that read-out positive or negative fluorescence signals (Figure 2D). The resulting digital data is then processed by a Poisson probability distribution to derive copy numbers. In fact, we previously showed that ddPCR can be a robust method that accurately detects the copy number of a retrotransposon (Fan and Cho, 2021). Importantly, the ddPCR technique only requires a trace amount of DNA and is therefore possible to be performed in DNAs extracted from small amount of tissues and rare samples.

### Population Level

#### Next-Generation Sequencing-Based Transposable Element Mapping

*Arabidopsis* 1,001 genome project produced massive paired-end short-read whole-genome sequencing data from 1,135 accessions from a worldwide collection (Weigel and Mott, 2009; Cao et al., 2011; Alonso-Blanco et al., 2016). Similar attempt has been made in rice generating sequencing data from a total of more than 3,000 germplasm accessions (Li J.-Y. et al., 2014; Li Z. et al., 2014). Equipped with relatively well-assembled and annotated reference genomes available for both plant species, TE insertion polymorphisms have been intensively profiled at population level. Several softwares have been developed so far to systematically identify transposon insertions. These tools take advantage of diverse sequencing read information; for instance, split reads in Transposon Insertion Finder (TIF) (Nakagome et al., 2014), SPLITREADER (Baduel et al., 2021b), and RTRIP (Liu et al., 2020), discordant read pair alignment in TRACKPOSON (Carpentier et al., 2019), and combination of these two as demonstrated in TEPID (Stuart et al., 2016). Additionally, in a recent work of Baduel et al. (2021a) SPLITREADER and TEPID pipelines were integrated, building an intensive map of TE landscape in *Arabidopsis*.

The split-reads method first searches for reads containing the end sequences of a TE and target site duplications (TSDs), which are identical sequences flanking a TE and created as a result of transposition (Figure 2E). In TIF, the read sequences tagged with transposon end sequences are mapped to the reference genome to identify the locations of *de novo* insertions (Nakagome et al., 2014). Similarly, SPLITREADER extracts reads that do not properly map to the reference genome and forcedly map to 5′ and 3′ TE sequence extremities (within a range of 300 bp) by soft clipping (Baduel et al., 2021b). Then, the bona fide insertions and their locations are identified by mapping the clipped reads to the reference genome. Recently, Liu et al. (2020) tested a similar method in rice and generated the RTRIP database, which contains the comprehensive profile of transposon insertion polymorphisms in the rice 3K genome project.

The discordant read pair method employs mapping of reads from one side to the target TE and the other side to a distant genomic region (Figure 2F). TRACKPOSON, for instance, first maps all reads of a given accession onto each TE family represented by a single consensus sequence, and then maps the unmapped paired reads to the rice reference genome to determine its location (Carpentier et al., 2019). The transposition landscape revealed by these methods uncovered that transposon proliferation is most strongly associated with the presence of a transposon at a specific location, which was somehow activated during the evolutionary process (Carpentier et al., 2019).

## Concluding Remarks and Future Perspectives

We reviewed the recent technical advances in transposon research by highlighting several new methods identifying active TEs and detecting transposition events (Figure 2). These novel experimental methods and improved transposon annotation aided by the long-read sequencing technologies and population-scale genome resequencing databases will open-up a new window to unveil a long-lasting mystery of jumping genes. Although the experimental techniques described above has greatly improved our ability to observe transposition events, there are still several issues left to be dealt with. Firstly, detection of transposition events at single-cell level will be obviously the next task to be accomplished. To this end, a novel approach for the single-cell detection of transposon mobilization is highly desired. Secondly, the single-cell genomics will vastly benefit the transposon biology. The transposition reporter systems introduced above rely on the artificially engineered TE sequences. The investigation of the native TEs and their transposition at high resolution will only be possible when the single-cell genomics technologies become more available. Thirdly, the detection sensitivity of transposon research tools will have to be improved further. The new experimental tools to study transposon such as ALE-seq and mobilome-seq are mostly tested in the epigenetic mutants where transposons become unusually active in mobility. Although these methods were sensitive enough to discover novel active retroelements (*Go-on* and *PopRice*), the moderately active TEs were difficult to be identified (Lanciano et al., 2017; Cho et al., 2019). Considering the rarity of DNAs representing activated TE intermediates or derived from transposed copy, the improvement of detection sensitivity of these methods will help identify new transposons that are present in small niches of cells or activated only to a moderate level. Altogether, the technical advances of transposon research at varying scales have greatly contributed to our understanding of TE life cycle and will broaden the breadth of knowledge on mobile genetic elements and genome plasticity.

## Author Contributions

WF, LW, JiC, HL, and EK drafted the manuscript. EK and JuC edited the manuscript. JuC revised the manuscript. All authors contributed to the article and approved the submitted version.

## Conflict of Interest

The authors declare that the research was conducted in the absence of any commercial or financial relationships that could be construed as a potential conflict of interest.

## Publisher’s Note

All claims expressed in this article are solely those of the authors and do not necessarily represent those of their affiliated organizations, or those of the publisher, the editors and the reviewers. Any product that may be evaluated in this article, or claim that may be made by its manufacturer, is not guaranteed or endorsed by the publisher.

## References

[B1] Alonso-Blanco C. Andrade J. Becker C. Bemm F. Bergelson J. Borgwardt K. M. (2016). 1,135 genomes reveal the global pattern of polymorphism in *Arabidopsis thaliana*. *Cell* 166 481–491. 10.1016/j.cell.2016.05.063 27293186 PMC4949382

[B2] Baduel P. Quadrana L. Colot V. (2021b). Efficient detection of transposable element insertion polymorphisms between genomes using short-read sequencing data. *Methods Mol. Biol.* 2250 157–169. 10.1007/978-1-0716-1134-0_15 33900602

[B3] Baduel P. Leduque B. Ignace A. Gy I. Gil J. Loudet O. (2021a). Genetic and environmental modulation of transposition shapes the evolutionary potential of *Arabidopsis thaliana*. *Genome Biol.* 22 138. 10.1186/s13059-021-02348-5 33957946 PMC8101250

[B4] Boeke J. D. Garfinkel D. J. Styles C. A. Fink G. R. (1985). Ty elements transpose through an RNA intermediate. *Cell* 40 491–500. 10.1016/0092-8674(85)90197-7 2982495

[B5] Bubner B. Baldwin I. T. (2004). Use of real-time PCR for determining copy number and zygosity in transgenic plants. *Plant Cell Rep.* 23 263–271. 10.1007/s00299-004-0859-y 15368076

[B6] Bubner B. Gase K. Baldwin I. T. (2004). Two-fold differences are the detection limit for determining transgene copy numbers in plants by real-time PCR. *BMC Biotechnol.* 4:14. 10.1186/1472-6750-4-14 15251044 PMC493272

[B7] Campomenosi P. Gini E. Noonan D. M. Poli A. D’Antona P. Rotolo N. (2016). A comparison between quantitative PCR and droplet digital PCR technologies for circulating microRNA quantification in human lung cancer. *BMC Biotechnol.* 16:60. 10.1186/s12896-016-0292-7 27538962 PMC4991011

[B8] Cao J. Schneeberger K. Ossowski S. Günther T. Bender S. Fitz J. (2011). Whole-genome sequencing of multiple *Arabidopsis thaliana* populations. *Nat. Genet.* 43 956–963. 10.1038/ng.911 21874002

[B9] Carpentier M.-C. Manfroi E. Wei F.-J. Wu H.-P. Lasserre E. Llauro C. (2019). Retrotranspositional landscape of Asian rice revealed by 3000 genomes. *Nat. Commun.* 10:24. 10.1038/s41467-018-07974-5 30604755 PMC6318337

[B10] Casacuberta J. M. Santiago N. (2003). Plant LTR-retrotransposons and MITEs: control of transposition and impact on the evolution of plant genes and genomes. *Gene* 311 1–11. 10.1016/S0378-1119(03)00557-2 12853133

[B11] Cho J. (2018). Transposon-derived Non-coding RNAs and their function in plants. *Front. Plant Sci.* 9:600. 10.3389/fpls.2018.00600 29774045 PMC5943564

[B12] Cho J. Benoit M. Catoni M. Drost H.-G. Brestovitsky A. Oosterbeek M. (2019). Sensitive detection of pre-integration intermediates of long terminal repeat retrotransposons in crop plants. *Nat. Plants* 5 26–33. 10.1038/s41477-018-0320-9 30531940 PMC6366555

[B13] Chuong E. B. Elde N. C. Feschotte C. (2017). Regulatory activities of transposable elements: from conflicts to benefits. *Nat. Rev. Genet.* 18 71–86. 10.1038/nrg.2016.139 27867194 PMC5498291

[B14] Cui Y. Barampuram S. Stacey M. G. Hancock C. N. Findley S. Mathieu M. (2012). Tnt1 retrotransposon mutagenesis: a tool for soybean functional genomics. *Plant Physiol.* 161 36–47. 10.1104/pp.112.205369 23124322 PMC3532266

[B15] Curcio M. J. Garfinkel D. J. (1991). Single-step selection for Ty1 element retrotransposition. *Proc. Natl. Acad. Sci. U.S.A.* 88 936–940. 10.1073/pnas.88.3.936 1846969 PMC50929

[B16] D’Erfurth I. Cosson V. Eschstruth A. Lucas H. Kondorosi A. Ratet P. (2003). Efficient transposition of the Tnt1 tobacco retrotransposon in the model legume *Medicago truncatula*. *Plant J.* 34 95–106. 10.1046/j.1365-313X.2003.01701.x 12662312

[B17] Doi H. Takahara T. Minamoto T. Matsuhashi S. Uchii K. Yamanaka H. (2015). Droplet digital polymerase chain reaction (PCR) outperforms real-time PCR in the detection of environmental DNA from an invasive fish species. *Environ. Sci. Technol.* 49 5601–5608. 10.1021/acs.est.5b00253 25850372

[B18] Dubin M. J. Mittelsten Scheid O. Becker C. (2018). Transposons: a blessing curse. *Curr. Opin. Plant Biol.* 42 23–29. 10.1016/j.pbi.2018.01.003 29453028

[B19] Esnault C. Maestre J. Heidmann T. (2000). Human LINE retrotransposons generate processed pseudogenes. *Nat. Genet.* 24 363–367. 10.1038/74184 10742098

[B20] Esposito S. Barteri F. Casacuberta J. Mirouze M. Carputo D. Aversano R. (2019). LTR-TEs abundance, timing and mobility in *Solanum commersonii* and *S. tuberosum* genomes following cold-stress conditions. *Planta* 250 1781–1787. 10.1007/s00425-019-03283-3 31562541

[B21] Fan W. Cho J. (2021). “Quantitative measurement of transposon copy number using the droplet digital PCR,” in *Plant Transposable Elements. Methods in Molecular Biology*, Vol. 2250 ed. Cho J. (New York, NY: Humana), 171–176. 10.1007/978-1-0716-1134-0_16 33900603

[B22] Feschotte C. (2008). Transposable elements and the evolution of regulatory networks. *Nat. Rev. Genet.* 9 397–405. 10.1038/nrg2337 18368054 PMC2596197

[B23] Głowacka K. Kromdijk J. Leonelli L. Niyogi K. K. Clemente T. E. Long S. P. (2016). An evaluation of new and established methods to determine T-DNA copy number and homozygosity in transgenic plants. *Plant Cell Environ.* 39 908–917. 10.1111/pce.12693 26670088 PMC5021166

[B24] Goodier J. L. Zhang L. Vetter M. R. Kazazian H. H. (2007). LINE-1 ORF1 protein localizes in stress granules with other RNA-binding proteins, including components of RNA interference RNA-induced silencing complex. *Mol. Cell. Biol.* 27 6469–6483. 10.1128/mcb.00332-07 17562864 PMC2099616

[B25] Grandbastien M. A. (2015). LTR retrotransposons, handy hitchhikers of plant regulation and stress response. *Biochim. Biophys. Acta Gene Regul. Mech.* 1849 403–416. 10.1016/j.bbagrm.2014.07.017 25086340

[B26] Griffiths J. Catoni M. Iwasaki M. Paszkowski J. (2018). Sequence-independent identification of active LTR Retrotransposons in *Arabidopsis*. *Mol. Plant* 11 508–511. 10.1016/j.molp.2017.10.012 29107035

[B27] Heidmann T. Heidmann O. Nicolas J. F. (1988). An indicator gene to demonstrate intracellular transposition of defective retroviruses. *Proc. Natl. Acad. Sci. U.S.A.* 85 2219–2223. 10.1073/pnas.85.7.2219 2832848 PMC279961

[B28] Heldmann O. Heidmann T. (1991). Retrotransposition of a mouse IAP sequence tagged with an indicator gene. *Cell* 64 159–170. 10.1016/0092-8674(91)90217-M 1846087

[B29] Hindson C. M. Chevillet J. R. Briggs H. A. Gallichotte E. N. Ruf I. K. Hindson B. J. (2013). Absolute quantification by droplet digital PCR versus analog real-time PCR. *Nat. Methods* 10 1003–1005. 10.1038/nmeth.2633 23995387 PMC4118677

[B30] Hirochika H. (2010). Insertional mutagenesis with Tos17 for functional analysis of rice genes. *Breed. Sci.* 60 486–492. 10.1270/jsbbs.60.486 26081539

[B31] Hirochika H. Sugimoto K. Otsuki Y. Tsugawa H. Kanda M. (1996). Retrotransposons of rice involved in mutations induced by tissue culture. *Proc. Natl. Acad. Sci. U.S.A.* 93 7783–7788. 10.1073/pnas.93.15.7783 8755553 PMC38825

[B32] Hung Y.-H. Slotkin R. K. (2021). The initiation of RNA interference (RNAi) in plants. *Curr. Opin. Plant Biol.* 61 102014. 10.1016/j.pbi.2021.102014 33657510

[B33] Jensen S. A. Heidmann T. (1991). An indicator gene for detection of germline retrotransposition in transgenic *Drosophila* demonstrates RNA-mediated transposition of the LINE I element. *EMBO J.* 10 1927–1937. 10.1002/j.1460-2075.1991.tb07719.x 1710982 PMC452868

[B34] Kim E. Y. Wang L. Lei Z. Li H. Fan W. Cho J. (2021b). Ribosome stalling and SGS3 phase separation prime the epigenetic silencing of transposons. *Nat. Plants* 7 303–309. 10.1038/s41477-021-00867-4 33649597

[B35] Kim E. Y. Fan W. Cho J. (2021a). Determination of TE insertion positions using transposon display. *Methods Mol. Biol.* 2250 115–121. 10.1007/978-1-0716-1134-0_11 33900598

[B36] Lanciano S. Carpentier M.-C. Llauro C. Jobet E. Robakowska-Hyzorek D. Lasserre E. (2017). Sequencing the extrachromosomal circular mobilome reveals retrotransposon activity in plants. *PLoS Genet.* 13:e1006630. 10.1371/journal.pgen.1006630 28212378 PMC5338827

[B37] Lee S. C. Ernst E. Berube B. Borges F. Parent J.-S. Ledon P. (2020). Arabidopsis retrotransposon virus-like particles and their regulation by epigenetically activated small RNA. *Genome Res.* 30 576–588. 10.1101/gr.259044.119 32303559 PMC7197481

[B38] Li J.-Y. Wang J. Zeigler R. S. (2014). The 3,000 rice genomes project: new opportunities and challenges for future rice research. *Gigascience* 3:8. 10.1186/2047-217X-3-8 24872878 PMC4035671

[B39] Li Z. Fu B. Y. Gao Y. M. Wang W. S. Xu J. L. Zhang F. (2014). The 3,000 rice genomes project. *Gigascience* 3:7. 10.1186/2047-217X-3-7 24872877 PMC4035669

[B40] Lisch D. (2012). How important are transposons for plant evolution? *Nat. Rev. Genet.* 14 49–61. 10.1038/nrg3374 23247435

[B41] Liu Z. Wang T. Wang L. Zhao H. Yue E. Yan Y. (2020). RTRIP: a comprehensive profile of transposon insertion polymorphisms in rice. *Plant Biotechnol. J.* 18 2379–2381. 10.1111/pbi.13425 32473053 PMC7680536

[B42] Long D. Martin M. Sundberg E. Swinburne J. Puangsomlee P. Coupland G. (1993). The maize transposable element system Ac/Ds as a mutagen in *Arabidopsis*: identification of an albino mutation induced by Ds insertion. *Proc. Natl. Acad. Sci. U.S.A.* 90 10370–10374. 10.1073/pnas.90.21.10370 8234300 PMC47776

[B43] Maestre J. Tchénio T. Dhellin O. Heidmann T. (1995). mRNA retroposition in human cells: processed pseudogene formation. *EMBO J.* 14 6333–6338. 10.1002/j.1460-2075.1995.tb00324.x 8557053 PMC394758

[B44] Martínez G. Slotkin R. K. (2012). Developmental relaxation of transposable element silencing in plants: functional or byproduct? *Curr. Opin. Plant Biol.* 15 496–502. 10.1016/j.pbi.2012.09.001 23022393

[B45] Mathieu M. Winters E. K. Kong F. Wan J. Wang S. Eckert H. (2009). Establishment of a soybean (*Glycine max* Merr. L) transposon-based mutagenesis repository. *Planta* 229 279–289. 10.1007/s00425-008-0827-9 18855007

[B46] Matzke M. A. Mosher R. A. (2014). RNA-directed DNA methylation: an epigenetic pathway of increasing complexity. *Nat. Rev. Genet.* 15 394–408. 10.1038/nrg3683 24805120

[B47] Mirouze M. Reinders J. Bucher E. Nishimura T. Schneeberger K. Ossowski S. (2009). Selective epigenetic control of retrotransposition in *Arabidopsis*. *Nature* 461 427–430. 10.1038/nature08328 19734882

[B48] Møller H. D. Larsen C. E. Parsons L. Hansen A. J. Regenberg B. Mourier T. (2016). Formation of extrachromosomal circular DNA from long terminal repeats of retrotransposons in *Saccharomyces cerevisiae*. *G3* 6 453–462. 10.1534/g3.115.025858 26681518 PMC4751563

[B49] Møller H. D. Parsons L. Jørgensen T. S. Botstein D. Regenberg B. (2015). Extrachromosomal circular DNA is common in yeast. *Proc. Natl. Acad. Sci. U.S.A.* 112 3114–3122. 10.1073/pnas.1508825112 26038577 PMC4475933

[B50] Moran J. V. Holmes S. E. Naas T. P. Deberardinis R. J. Boeke J. D. Kazazian H. H. (1996). High frequency retrotransposition inin cultured mammalian cells. *Cell* 87 917–927. 8945518 10.1016/s0092-8674(00)81998-4

[B51] Nakagome M. Solovieva E. Takahashi A. Yasue H. Hirochika H. Miyao A. (2014). Transposon insertion finder (TIF): a novel program for detection of de novo transpositions of transposable elements. *BMC Bioinformatics* 15:71. 10.1186/1471-2105-15-71 24629057 PMC4004357

[B52] Nandety R. S. Serrani-Yarce J. C. Gill U. S. Oh S. Lee H. Zhang X. (2020). Insertional mutagenesis of *Brachypodium distachyon* using the Tnt1 retrotransposable element. *Plant J.* 103 1924–1936. 10.1111/tpj.14813 32410353 PMC7496502

[B53] Ostertag E. M. Luning Prak E. T. DeBerardinis R. J. Moran J. V. Kazazian H. H. (2000). Determination of L1 retrotransposition kinetics in cultured cells. *Nucleic Acids Res.* 28 1418–1423. 10.1093/nar/28.6.1418 10684937 PMC111040

[B54] Panda K. Slotkin R. K. (2020). Long-Read cDNA sequencing enables a “Gene-Like” transcript annotation of transposable elements. *Plant Cell* 32 2687–2698. 10.1105/tpc.20.00115 32647069 PMC7474280

[B55] Rangwala S. H. Kazazian H. H. (2009). The L1 retrotransposition assay: a retrospective and toolkit. *Methods* 49 219–226. 10.1016/j.ymeth.2009.04.012 19398011 PMC2763987

[B56] Reinders J. Paszkowski J. (2009). Unlocking the *Arabidopsis* epigenome. *Epigenetics* 4 557–563. 10.4161/epi.4.8.10347 19934651

[B57] Reinders J. Wulff B. B. H. Mirouze M. Mari-Ordonez A. Dapp M. Rozhon W. (2009). Compromised stability of DNA methylation and transposon immobilization in mosaic *Arabidopsis* epigenomes. *Genes Dev.* 23 939–950. 10.1101/gad.524609 19390088 PMC2675864

[B58] Revalska M. Vassileva V. Goormachtig S. Van Hautegem T. Ratet P. Iantcheva A. (2011). Recent progress in development of Tnt1 functional genomics platform for *Medicago truncatula* and *Lotus japonicus* in Bulgaria. *Curr. Genomics* 12 147–152. 10.2174/138920211795564313 21966253 PMC3129049

[B59] Roquis D. Robertson M. Yu L. Thieme M. Julkowska M. Bucher E. (2021). Genomic impact of stress-induced transposable element mobility in *Arabidopsis*. *Nucleic Acids Res.* 49 10431–10447. 10.1093/nar/gkab828 34551439 PMC8501995

[B60] Satheesh V. Fan W. Chu J. Cho J. (2021). Recent advancement of NGS technologies to detect active transposable elements in plants. *Genes Genomics* 43 289–294. 10.1007/s13258-021-01040-z 33555503

[B61] Slotkin R. K. Martienssen R. (2007). Transposable elements and the epigenetic regulation of the genome. *Nat. Rev. Genet.* 8 272–285. 10.1038/nrg2072 17363976

[B62] Stuart T. Eichten S. R. Cahn J. Karpievitch Y. V. Borevitz J. O. Lister R. (2016). Population scale mapping of transposable element diversity reveals links to gene regulation and epigenomic variation. *Elife* 5:e20777. 10.7554/eLife.20777 27911260 PMC5167521

[B63] Tadege M. Wen J. He J. Tu H. Kwak Y. Eschstruth A. (2008). Large-scale insertional mutagenesis using the Tnt1 retrotransposon in the model legume *Medicago truncatula*. *Plant J.* 54 335–347. 10.1111/j.1365-313X.2008.03418.x 18208518

[B64] Tchenio T. Heidmann T. (1992). High-frequency intracellular transposition of a defective mammalian provirus detected by an in situ colorimetric assay. *J. Virol.* 66 1571–1578. 10.1128/jvi.66.3.1571-1578.1992 1371167 PMC240883

[B65] Tchenio T. Segal-Bendirdjian E. Heidmann T. (1993). Generation of processed pseudogenes in murine cells. *EMBO J.* 12 1487–1497. 10.1002/j.1460-2075.1993.tb05792.x 8385606 PMC413361

[B66] Tenaillon M. I. Hollister J. D. Gaut B. S. (2010). A triptych of the evolution of plant transposable elements. *Trends Plant Sci.* 15 471–478. 10.1016/j.tplants.2010.05.003 20541961

[B67] Thieme M. Lanciano S. Balzergue S. Daccord N. Mirouze M. Bucher E. (2017). Inhibition of RNA polymerase II allows controlled mobilisation of retrotransposons for plant breeding. *Genome Biol.* 18 134. 10.1186/s13059-017-1265-4 28687080 PMC5501947

[B68] Wang L. Cho J. Satheesh V. (2021a). Bioinformatics analysis guides to LTR retrotransposon-derived extrachromosomal linear DNAs identified by ALE-seq. *Methods Mol. Biol.* 2250 111–114. 10.1007/978-1-0716-1134-0_10 33900597

[B69] Wang L. Kim E. Y. Cho J. (2021b). High-throughput profiling of extrachromosomal linear DNAs of long terminal repeat retrotransposons by ALE-seq. *Methods Mol. Biol.* 2250 103–110. 10.1007/978-1-0716-1134-0_9 33900596

[B70] Wang N. Long T. Yao W. Xiong L. Zhang Q. Wu C. (2013). Mutant resources for the functional analysis of the rice genome. *Mol. Plant* 6 596–604. 10.1093/mp/sss142 23204502

[B71] Weigel D. Mott R. (2009). The 1001 genomes project for *Arabidopsis thaliana*. *Genome Biol.* 10:107. 10.1186/gb-2009-10-5-107 19519932 PMC2718507

[B72] Xie Y. Rosser J. M. Thompson T. L. Boeke J. D. An W. (2011). Characterization of L1 retrotransposition with high-throughput dual-luciferase assays. *Nucleic Acids Res.* 39:e16. 10.1093/nar/gkq1076 21071410 PMC3035435

